# Maxillary Antrostomy Patency Following Intraoperative Use of Spray Cryotherapy

**DOI:** 10.3390/jcm9010088

**Published:** 2019-12-29

**Authors:** Veronica Trombitaș, Adriana Zolog, Mioriţa Toader, Silviu Albu

**Affiliations:** 1 II-nd Department of Otolaryngology, Iuliu Hatieganu University of Medicine and Pharmacy, Str. Republicii nr. 18, 400015 Cluj-Napoca, Romania; silviualbu63@gmail.com; 2 Pathology Department, CF Hospital Cluj-Napoca, 400015 Cluj-Napoca, Romania; adizolog@yahoo.com; 3 Department of Otolaryngology, Grigore Alexandrescu Hospital, 011743 Bucuresti, Romania; toadermiorita@yahoo.com

**Keywords:** middle meatus antrostomy, endoscopic sinus surgery, spray cryotherapy, nasal mucosa, histology

## Abstract

**Objectives**/**Hypothesis:** Stenosis of the middle meatus antrostomy (MMA) represents a major cause of recurrent disease following endoscopic sinus surgery (ESS). Various strategies have been developed to prevent the occurrence of MMA stenosis. The aim of the present study was to evaluate the effects of spray cryotherapy (SC) on nasal wound healing following ESS. **Methods:** This is a prospective within-subject, randomized, and controlled trial. Twenty-six patients submitted to bilateral ESS with chronic rhinosinusitis without polyps were included. Following surgery, patients were randomized to receive SC on one side and saline contralaterally. Outcomes were represented by MMA diameter and area, histology of nasal mucosa, and nasal symptoms. Variables were assessed at 3 and 12 months postoperatively. **Results:** The MMA size in the SC group at 3 and 12 months (area—0.578 ± 0.1025 cm^2^, diameter—0.645 ± 0.1024 cm; 0.605 ± 0.1891 cm^2^, 0.624 ± 0.0961 cm, respectively) was significantly larger (*p* = 0.000) than in the control group. Histology established that cell infiltration, goblet cells, edema, and epithelial hyperplasia were prominent and persistent in the control side compared to the SC side. Nasal obstruction and discharge were significantly improved in the SC group compared to the control group. **Conclusion:** SC is a promising therapy following ESS, since it precludes MMA stenosis and decreases inflammation, edema, and goblet cell hyperplasia.

## 1. Introduction

Endoscopic sinus surgery (ESS) represents the treatment of choice for chronic rhinosinusitis (CRS) which is unresponsive to maximal medical therapy [1,2,3,4,5]. Recently, ESS has been used extensively in the management of odontogenic CRS and for the extraction of foreign bodies, impacted teeth, and dentigerous cysts from the maxillary sinus [6,7]. Approximately 10%–30% of CRSs are due to a dental source [6]. Recently, Trimarchi et al. described the successful management of dental CRS with the combined transoral (root canal therapy, dental extraction, dental implant removal, oro-antral fistula closure) and ESS approaches [6]. Today, it is widely acknowledged that dental implantation with or without sinus lifting requires close cooperation between otorhinolaryngologists (ENT) and dentists. ENT surgeons are not only involved in the treatment of post-implant CRS, but mainly in the pre-implant management in order to prevent the development of sinus complications [8,9,10,11]. Although ESS is highly successful, failures are still reported in 15% to 26% of patients [12,13,14]. Adhesions and stenosis of the middle meatus antrostomy (MMA) are intensely associated with unsuccessful ESS outcomes [13,14]. Adhesion and MMA stenoses occur as a result of scars and/or granulation development during the tissue recovery process [12,13,14]. Several substances, such as mitomycin and vitamin A, were used in clinical trials to impede development of adhesions [15,16,17].

Endoscopic spray cryotherapy (SC) using low-pressure liquid nitrogen is a new method successfully used in dermatology, oncology, ophthalmology, and gastroenterology [18,19,20,21,22,23,24]. SC with liquid nitrogen (−196 °C) is a noncontact method of tissue ablation. SC destroys tissues, interrupts the vascular supply, and stimulates the immune system [19]. In addition, SC protects the tissue architecture and extracellular matrix, which remain largely intact, generating a favorable wound response [20,21]. SC has also been successfully employed during bronchoscopy, followed or not by balloon dilatation [22,23]. In otolaryngology, SC has been used in patients with glottic and subglottic benign stenoses [24].

We have recently demonstrated that SC significantly improved postoperative objective scores and enhanced healing following ESS [25]. In another paper, we demonstrated that SC increased the antrostomy patency in a CRS rabbit model [26]. In this study, we aimed to assess the consequences of SC on MMA patency. In addition, subjective outcomes were also determined and correlated with the histological pattern.

## 2. Materials and Methods

**Ethical considerations.** The study was approved by the Ethic Committee of the Iuliu Hateganu University of Medicine and Pharmacy Cluj-Napoca (No.10/16January 2014). All patients have signed an informed consent preceding inclusion in the study. 

**Inclusion criteria.** This study was a prospective, randomized, and controlled trial. We included the patients presenting bilateral CRS without polyps (CRSsNP) according to the criteria endorsed in the European Position Paper on Rhinosinusitis and Nasal Polyps 2012 [1]. Each patient underwent a preoperative computed tomography (CT) scan, and disease severity was graded according to the Lund–Mackay (LM) system [27]. Exclusion criteria included patients with a difference between sides of higher than two grades in the LM system, prior nasal surgery, nasal polyps, unilateral disease, severe septal deformity, cystic fibrosis, aspirin intolerance, immotile cilia syndrome, neutropenia, and patients with recognized autoimmune diseases [5,15,16,17,28]. Likewise, smokers and patients with bronchial asthma were excluded from the present study. 

**Surgery.** All patients were operated on under general anesthesia by the same senior surgeon. The amount of sinus disease entailed the extent of the surgery fulfilled in each patient [29]. However, every patient included in the study went through uncinectomy, MMA, and anterior ethmoidectomy. MMA was performed using standard instrumentation—using cutting and backbiting forceps, enlarging its diameter in the anterior and posterior directions. When a septal spur restricted the surgical procedure, a septoplasty was also achieved. The middle turbinate was preserved and a mucosa-sparing technique was employed in all patients. Since liquid nitrogen was sprayed close to the orbit, we implemented several strategies to prevent orbital complications. Backbiting forceps were used to perform the uncinectomy, and the lamina papyracea was identified early. The performance of a large MMA permitted the achievement of the three dimensional perspective, allowing one to simultaneously foresee the orbital floor, medial orbital wall, and posterior maxillary wall. The lamina papyracea was skeletonized early during ethmoidectomy to prevent orbital penetration. The removal of all bony septations allowed complete exposure of the lamina.

**Figure 1:**
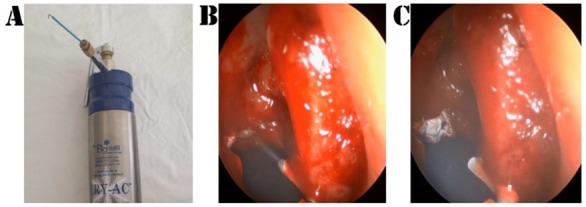
(**A**) Device of spray cryotherapy. (**B**,**C**) Spray cryotherapy application.

Patients were randomized by means of a closed envelope system: SC was conveyed in one meatus and saline in the contralateral side. At the conclusion of the procedure, the envelope was unlocked and SC or saline were delivered over the boundaries of the MMA according to the randomization. SC was performed with the Brymill CRY-AC^®^-3 Cryogenic System (Figure 1) and consisted of 4 sequences of gas delivery (each cycle lasting 5 s) with breaks of 35 s for a complete thaw of the tissues [25]. Mucosa samples were collected from the edges of the MMA for histological evaluation (see Table 1).

At the end of the procedures, both sides were packed with Vaseline gauze. Packing was removed the following day, and patients were discharged on oral broad-spectrum antibiotics for 10 days and daily saline nasal irrigation for 4 weeks (twice a day). 

**Figure 2:**
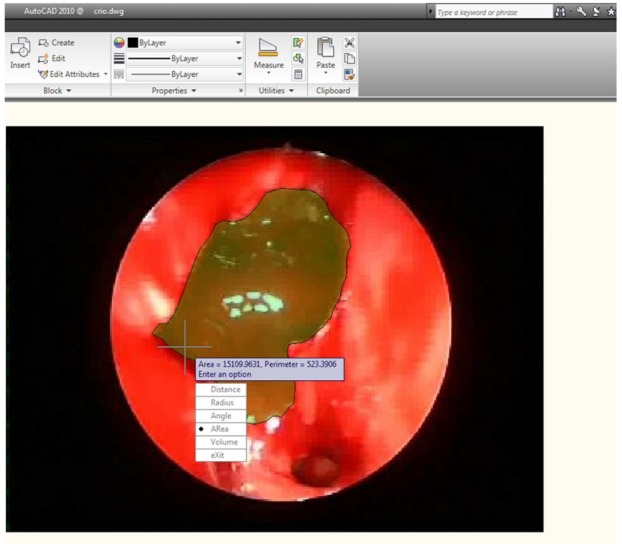
AutoCAD evaluation of maxillary antrostomy.

**Figure 3:**
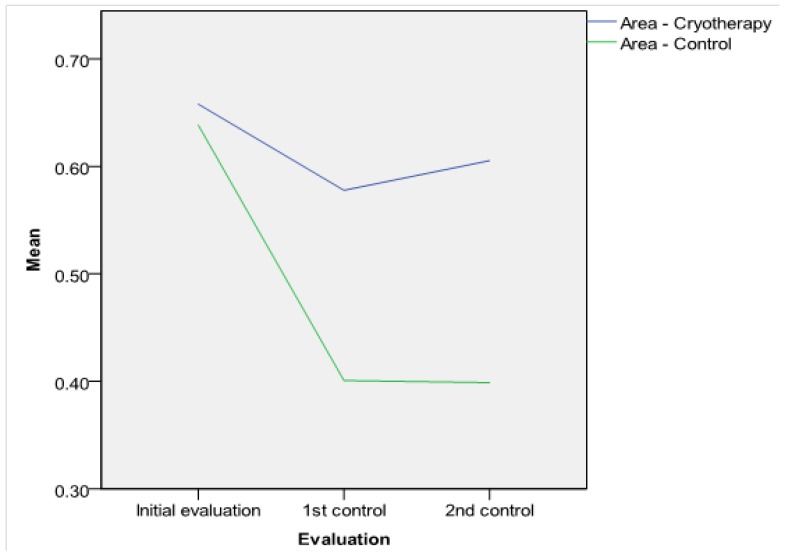
Middle meatus antrostomy area—comparison between the two groups.

**Figure 4:**
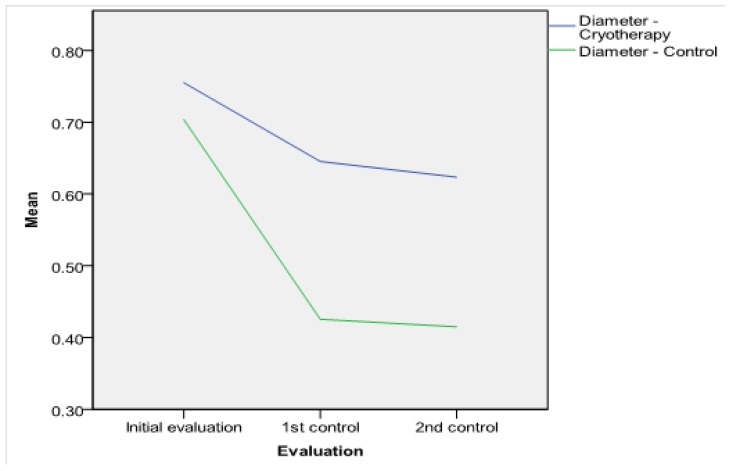
Middle meatus antrostomy diameter—comparison between the two groups.

**Outcome measurement.** Postoperative outcomes were assessed by an investigator blinded to the surgical procedure at 3 months (1st follow-up visit) and 12 months (2nd follow-up visit) following ESS. During each follow-up visit, each nostril was scrutinized by means of a 30° and 45° angled endoscope. Accurate and reproducible measurements of the MMA were obtained according to the previously published accepted guidelines [15,17]: A circular probe was positioned in the same plane as the MMA and in the center of the endoscope’s field of view. When the contour of the probe in the endoscope’s field of view was as spherical as possible, the probe was withdrawn and freeze-frame shots were taken (Figure 2). The MMA areas and diameters were objectively measured using the AutoCAD 2010 (Autodesk Inc., San Rafael, CA, USA) system (Figure 3 and Figure 4). Tissue biopsies were taken from the MMA area during the operative procedure and during the follow-up. Tissue samples were fixed in formaldehyde and stained with hematoxylin and eosin, as well as Masson Trichrome for collagen. A single senior pathologist evaluated the mucosa samples. The histological assessment included the presence of mononuclear cell infiltrate, edema, cilia characteristics, grade of fibrosis, number of goblet cells, epithelial hyperplasia, and squamous metaplasia (see Table 1). All of the mentioned parameters were recorded as categorical variables.

Patient symptoms (nasal blockage, nasal discharge, facial pressure) were recorded before surgery and at the follow-up visits for both operated sides. Patients scored these symptoms on a visual analogue scale (VAS) scale, where absent = 0 and severe = 10. Periorbital swelling and pain, proptosis, globe displacement, eye movements, and visual acuity were assessed preoperatively and compared with postoperative outcomes.

**Statistical Analysis**. Data are presented as mean ± SD. In a previous investigation, the mean MMA diameter at 3 months was 0.44 cm ± 0.15 cm [24]. The sample size was determined in the following manner: Assuming a 20% difference between the two sides, a power of 80%, and a significance level of 5%, the sample size comprised 23 patients according to the McNemar test. To compensate for drop-outs, the number was increased to 26. The results were analyzed using SPSS Statistics version 20.0 for Windows (SPSS Inc., Chicago, IL, USA). In order to evaluate the normal distribution of data, the Kolmogorov–Smirnov test was used. For normal distributed parameters, the Student t-test and the ANOVA analysis were used for comparison. When ordinal or non-normally distributed data were assessed, we employed the Mann–Whitney U and Kruskall–Wallis tests, as well as the Kendall’s correlation coefficient in the tau-b form. The Spearman rank correlation test was used for correlations. The Wilcoxon test was used for comparison of matched pairs. A receiver operating characteristic (ROC) curve analysis was performed to evaluate the effect of SC on MMA patency. In all analyses, *p*-values were evaluated for the 1%, 5%, and 10% significance values, depending on the probabilities obtained.

## 3. Results

**Patients.** In the present study, 26 patients (11 males/15 females) ranging in age from 21 to 74 years (mean 44.5 years) were enrolled. The mean (±SD) preoperative LM score in the SC-treated side was 5.5 ± 1.2, and 6.2 ± 1.8 in the control side (*p* > 0.05). No lamina papyracea dehiscence was noted on the coronal preoperative CT scan in any patient included in the study. No major complications (orbital hematoma, visual loss, significant hemorrhage requiring transfusion, CSF leaks, meningitis, or brain abscess) were noted in our cohort. Since the lamina papyracea was kept intact in all our cases, no subcutaneous emphysemas or periorbital ecchymoses were encountered. However, minor complications were noted: One postoperative epistaxis (control side) managed with nasal packing and one postoperative sinus infection (control side) managed with antibiotics.

**Figure 5:**
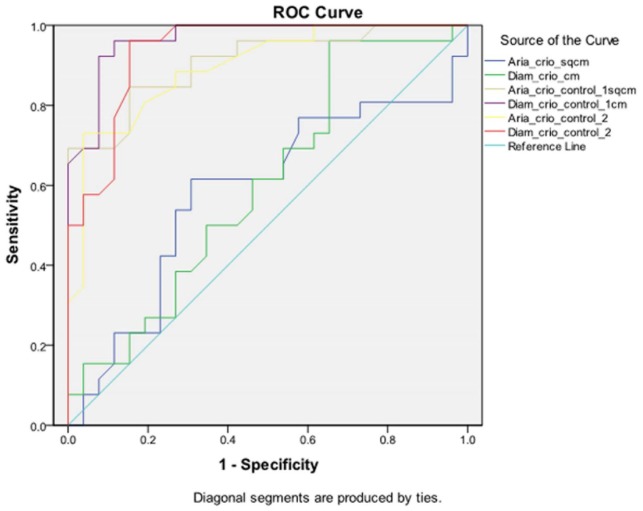
ROC curve.

**Figure 6:**
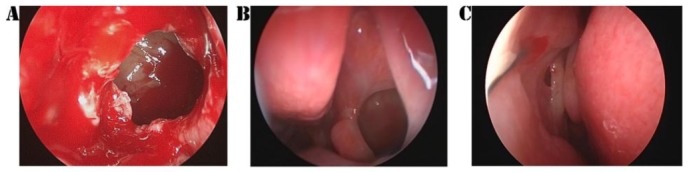
The evolution of the middle meatus antrostomy. (**A**) Intraoperative endoscopic aspect. (**B**) Spray cryotherapy—second follow-up endoscopic aspect. (**C**) Control—second follow-up endoscopic aspect.

**Antrostomy dimensions.** The intraoperative MMA areas and diameters were similar in both groups (*p* > 0.05, see Table 2). The postoperative MMA was larger in the SC-treated side in comparison with the control side: On the first follow-up visit, the differences in diameter and area between the two groups were statistically significant (see Table 2). At 12 months, the difference was significant at the 1% critical limit (*p* = 0.000 < 0.01, see Table 2). The comparison of both MMA areas and diameters in the two groups is represented in Figure 3 and Figure 4. The results of the t-test were certified in the ROC investigation (see Table 3). Thus, at the initial assessment, there are no differences in the area under the curve (AUC). However, examination at 3 and 12 months reveals that differences in the AUC between the groups attain significance at a 1% value. The ROC curve is presented in Figure 5. According to previous data [30], MMA stenosis was considered when the diameter was less than 6 mm. At the final follow-up, we encountered one MMA with a reduced dimension in an SC patient and eight MMAs with stenoses in the control group (Figure 6). In the control group, five patients had stenoses rated as mild (diameter < 6 mm), two patients had stenoses rated as moderate (diameter < 5 mm), and one was rated as severe (diameter < 4 mm). In the treatment group, there was just one MMA with stenosis rated as mild.

**Figure 7:**
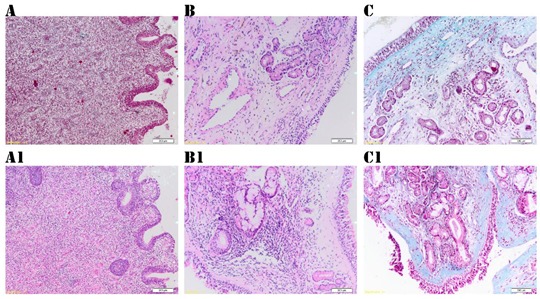
The histological examination of mucosa from the margins of the maxillary antrostomy. Spray cryotherapy (SC) group: (**A**) Three months following cryotherapy: Edematous stroma, minimal inflammatory infiltrate, epithelial hyperplasia (Hematoxylin and Eosin stain, 10×). (**B**) Histological aspect at 12 months after SC: Collagen fibers in the corium, sero glandular mucous acini, and excretory ducts (Hematoxylin and Eosin stain, 10×). (**C**) Minimal fibrosis in the corium at 12 months after SC (Masson Thrichrome stain, 10×). Control group: (**A1**) Three months following surgery: mucosa with reactive alterations, edema, important inflammatory infiltrate (Hematoxylin and Eosin stain, 10×). (**B1**) Twelve months following surgery: Mucosa with moderate edema, fibrosis, and inflammatory infiltrate (Hematoxylin and Eosin stain, 10×). (**C1**) Twelve months following surgery: Fibrosis and important epithelial hyperplasia (Masson Thrichrome stain, 10×).

**Histological analysis.** The histological analysis highlighted that the fibrosis, mononuclear cell infiltrate, edema, epithelial hyperplasia, and squamous metaplasia had similar scores in the two groups at surgery (see Table 4). At both the first and second follow-up visits, there was no statistical difference for the fibrosis scores between the two sides. Thus, the SC group had a minimal enhancement effect on fibrosis, but the power relationship was low (coefficient Kendall = 0.221 ∊ [0; 0.3], see Table 5). The mononuclear cell infiltrate, edema, goblet cell number, and epithelial hyperplasia were decreased in the SC group, and the difference was statistically significant during the follow-up (see Table 4 and Table 5). As clearly depicted in Table 5, at the first follow-up, the differences between the two groups were significant for edema (*p*-value = 0.000 < 0.01, coefficient Kendall = 0.543 ∊ [0.3; 0.7]), mononuclear cell infiltrate (*p*-value = 0.000 < 0.01, coefficient Kendall = −0.391 ∊ [0.3; 0.7]), and goblet cells (*p*-value = 0.000 < 0.01, coefficient Kendal = −0.422 ∊ [0.3; 0.7]). At the second follow-up, lessening of inflammation was obvious on both sides. However, mononuclear cell infiltrate was significantly reduced on the SC side at a threshold of statistical significance of 1%, the edema differences between the two sides were significant at a threshold of 5%, the goblet cell differences were significant at a threshold of 1%, and the ciliated cells displayed significant differences at a threshold of 1% (see Table 4 and Table 5). On the second follow-up, we concluded that mononuclear cell infiltrate (10–30 cells/field 40×), edema with collagen fiber dislocation, epithelial hyperplasia, and goblet cells (10–50 cells/field 40×) were more abundant on the control side (Figure 7). These histological features were related with MMA stenosis: There was a significant correlation between persistent inflammation and MMA dimensions (Table 6). Reduced MMA diameter and area were significantly associated with increased edema, epithelial hyperplasia, and increased numbers of goblet cells (Table 6 and Figure 7). An increased number of inflammatory cells was associated with a higher number of goblet cells and epithelial hyperplasia (Table 7 and Figure 7). Conversely, increased edema and hyperplasia of epithelium were associated with a higher number of goblet cells (Table 7).

On the SC side, we found a better organization of collagen fibers, the absence of epithelial hyperplasia, and that the number of goblet cells was statistically reduced (0–10 cells/field 40×). The squamous metaplasia persists only in the control group and in association with epithelial hyperplasia (Figure 7).

**Subjective outcomes.** When compared with the preoperative scores, at the last follow-up, the recorded symptoms demonstrated a significant reduction (*p* < 0.001, Wilcoxon test). Nasal obstruction and discharge were significantly improved in the SC group in comparison with the control group (see Table 8), while facial pressure scored similarly in both groups at the last follow-up. An increased number of goblet cells was associated with a greater postoperative obstruction (Spearman test, r = 0.412, *p* = 0.04) and discharge score (Spearman test, r = 0.452, *p* = 0.03). On the other hand, a reduced number of ciliated cells was associated with increased discharge (Spearman test, r = −0.442, *p* = 0.02).

Since liquid nitrogen was sprayed around the orbit, visual assessments were carefully recorded and compared with preoperative data and are listed in Table 9.

## 4. Discussion

Although credited with high success rates, suboptimal ESS outcomes could be attributed to different factors, such as persistent infection, massive inflammation, or poor surgical technique, all resulting in defective wound healing [1,2,3,4,5,6,7,8]. The Caldwell–Luc technique is no longer in use for the management of odontogenic CRS and foreign body extraction from the maxillary sinus due to its high morbidity [6,7]. Several recent papers describe the successful use of ESS in the management of odontogenic sinusitis [6,7,8,9,10,11]. In addition, an in-depth ENT evaluation is required prior to maxillary sinus elevation (particularly transcrestal sinus lifting) and dental implantation [8,9,10,11]. Appropriate sinus drainage and ventilation should be restored by means of medical or surgical therapy in advance of (before) dental management. ESS is considered the treatment of choice in several disorders that compromise maxillary ventilation: Anatomical variants impairing the patency of the osteomeatal complex, recurrent or chronic sinusitis, fungus ball, foreign bodies, and oroantral fistula. These illnesses should be submitted to ESS prior to or in association with maxillary elevation [6,7,8,9,10,11].

In addition to synechia, stenosis of the MMA is associated with poor outcomes following ESS. Stenosis of the maxillary ostium was reported to occur in 27% to 39% of revision ESS cases [6,7,8]. Musy and Kountakis [13] described common findings associated with primary ESS failure: Inadequate anterior ethmoidectomy (64%), scarring of the frontal recess (50%), and MMA stenosis (39%). In the cohort of 52 revision cases reported by Ramadan [12], residual disease in the ethmoid cells was the principal cause of recurrence, followed by MMA stenosis. In an attempt to increase MMA patency rates, various substances have been used in the clinical setting. Mitomycin C hinders fibroblast proliferation and migration, thus limiting adhesion formation [15,16]. Following topical mitomycin C application, MMA stenosis is significantly reduced. However, the drug is carcinogenic in rats and the long-term safety remains unknown [15,16]. Another recent study revealed that topical vitamin A is able to reduce adhesions and improve MMA patency rates following ESS [17]. These authors demonstrated that topical vitamin A hinders the proliferation and migration of fibroblasts. Most importantly, this study established that topical vitamin A treatment enhances the increase of ciliated cell numbers [17].

The present study aims to assess the efficacy of SC in maintaining the MMA patency. We used a device with an ambient pressure system, the solution was liquid nitrogen at −196 °C, and the pulverizations were imprinted around the maxillary antrostomy. In the human airways, cryotherapy was able to improve mucosal healing, as was clearly histologically confirmed [23,24]. The histologic characteristic was a better collagen organization and reduced keratinization [23,24].

In order to carefully assess the precise effect of SC on the healing of nasal mucosa, we followed strict inclusion and exclusion criteria [15,16,17,28]. In addition to the well-known, accepted, and published exclusion factors, we also rejected smokers and patients with asthma from the present investigation. Recent reviews have undoubtedly established that smoking adversely affects wound healing following ESS [31,32]. Asthma is clearly associated with poor objective and subjective postsurgical outcomes [28].

We were able to demonstrate a significant increase in both the MMA diameter and area on the SC side. During the follow-up visits, the MMA patency was constantly larger in the SC-treated group. The effect of increasing the MMA dimensions was also clearly confirmed by means of the ROC curve.

In an experimental model, we proved that cryotherapy is associated with reduced cellular inflammatory infiltrate and fibrosis [26]. On the other hand, SC enhances the development of ciliated cells and better collagen fiber organization [26].

Histology in our group of patients demonstrated that the mononuclear cell infiltration, goblet cells, edema, and epithelial hyperplasia were prominent and persistent in the control side compared to in the SC side. During the healing process, epithelial cells cooperate with inflammatory cells and release cytokines and growth factors, including transforming growth factor ß (TGF-ß) [32,33,34,35,36,37]. TGF-ß is an inflammation modulator and is considerably involved in extracellular matrix production in the respiratory system. Extracellular matrix deposition depends largely on the activity of local fibroblasts and myofibroblasts, and is induced primarily by local inflammatory factors such as interleukins (IL 10) and TGF-ß, produced mainly by M2 macrophages [35,36,37]. An improved collagen organization in the cryotreated side is related to the activity of effector T lymphocytes (TH1 and TH2) and mediators. Even if the local inflammation reaction is decreased in the cryotreated group, the increased M2 activity could explain the collagen deposition. Both clinical and experimental research proved that cryotherapy enhances collagen III production [26,35,36,37].

Another interesting finding was that small MMAs are significantly associated with important edema, epithelial hyperplasia, and increased goblet cells. In line with other published papers [32,33,34], we found that augmentation of inflammatory cells was associated with a higher number of goblet cells and epithelial hyperplasia. This finding is predictable, since the extracellular space significantly increases during inflammation [38,39,40].

Subjective outcomes underlined the efficacy of surgical treatment, since all patients had a clear improvement of SC symptoms. However, obstruction and discharge were significantly improved in the SC group compared to the controls. Increased obstruction and discharge were linked to higher numbers of goblet cells, and reduced numbers of ciliated cells accompanied increased discharge. Thus, persistent mucosal inflammation goes along with increased obstruction and discharge. Since SC significantly decreases tissue inflammation, the SC group subjectively fared better. Another factor associated with poor postsurgical outcomes and recurrent sinusitis is the presence of biofilms [41,42]. Even though the pathology of CRS is assumed to be multifactorial, biofilms are supposed to concur to persistent mucosal inflammation and recurrent disease [41,42]. Recently, an in vitro investigation demonstrated that SC is effective in disruption of biofilms in CRS [43]. Thus, the latter effect may also be responsible for the better outcome in our SC group of patients. According to current knowledge, it is reasonable to assume that proper surgical technique comprising ample opening of the maxillary ostium will suffice to maintain adequate MMA patency in the postoperative period. Thus, cryotherapy along with topical mitomycin C and topical vitamin A should be credited as an alternative method to keep the ostium open after inappropriate surgical techniques. However, MMA stenosis is still being reported as an essential finding in revision cases by well-known surgeons [12,13]. Attempts to maintain postsurgical patency should be considered in severe nasal polyposis with frequent relapses. Nevertheless, a recent investigation confirmed that SC in association with ESS improved objective and subjective outcomes in patients with nasal polyps [44]. Moreover, this study should be considered a prerequisite for the use of cryotherapy in the postoperative care of patients with Draf III procedures, where frontal sinus ostium stenosis is a major concern.

**Limitations.** The present study has several limitations: The restricted number of patients, IL 10 and TGF-ß were not objectively assessed, and the design of the study with patients acting as their own controls. Even if this strategy decreases biases emerging from natural differences among patients, it also has significant drawbacks: Persistent inflammation on one side may afflict the other nasal fossae, and quality of life (QoL) questionnaires cannot be used in the postoperative assessment [44]. It is widely acknowledged that QoL represents the gold standard in assessing subjective outcomes following ESS [44]. A longer follow-up is warranted to confirm our preliminary results.

**Strengths of this study**: Prospective, randomized, and controlled clinical study. Appropriate statistical methods used in validating the MMA size difference between the two groups. The present investigation assessed the histology pattern in SC-treated nasal mucosa.

**Clinical applicability of the study**: SC may represent a future therapy in preventing MMA stenosis and synechia following ESS. Although in most instances, correct surgical technique will result in adequate MMA size in the postoperative period, MMA stenosis is still reported in literature. In addition, mega-antrostomy and medial maxillectomy are employed in revision cases or benign tumors, not in primary CRS cases. Thus, SC may represent a future therapy in preventing MMA stenosis and synechia following ESS.

## Figures and Tables

**Table 1:** Inflammatory parameters used in the histological assessment [26].

Parameter	Grade	Description
**Mononuclear (inflammatory) cell infiltrate**	0	Normal aspect: 0–10 cells/HPF
	1	Discrete inflammation: 11–30 cells/HPF
	2	Moderate inflammation: 31–50 cells/HPF
	3	Severe inflammation: >50 cells/ HPF
**Edema**	0	No edema
	1	Focal sub-epithelial edema
	2	Diffuse sub-epithelial edema
	3	Diffuse sub-epithelial and intraglandular edema
**Cilia**	0	Normal aspect
	1	Shortened cilia
	2	Dotted cilia disappearance
	3	Lack of cilia
**Goblet cells**	0	Normal aspect: 0 cells/HPF
	1	Discrete inflammation: 0–30 cells/HPF
	2	Moderate inflammation: 31–50 cells/HPF
	3	Severe inflammation: >50 cells/HPF
**Fibrosis**	0	No fibrosis
	1	Sub-epithelial fibrosis
	2	Sub-epithelial and interglandular fibrosis
	3	Diffuse fibrosis (sub-epithelial and interglandular) with compression atrophy of glands and capillaries
**Epithelial hyperplasia**	0	No epithelial hyperplasia
	1	Dotted hyperplasia
	2	Diffuse hyperplasia
**Squamous metaplasia**	0	No squamous metaplasia
	1	Immature squamous metaplasia/dotted
	2	Mature squamous metaplasia/diffuse

HPF = high-pass filter, 40×.

**Table 2:** Area and diameter assessment in cryotherapy and control groups.

	Cryotherapy Side		Control Side	*p*-Value Student *t*-Test
	Mean ± SD		Mean ± SD	
Area (cm^2^)	0.658 ± 0.0998		0.638 ± 0.0872	0.456
Diameter (cm)	0.755 ± 0.1393		0.707 ± 0.1291	0.173
Area—1st follow-up visit (cm^2^)	0.578 ± 0.1025		0.401 ± 0.0804	0.000
Diameter—1st follow-up visit (cm)	0.645 ± 0.1024		0.426 ± 0.0828	0.000
Area—2nd follow-up visit (cm^2^)	0.605 ± 0.1891		0.399 ± 0.0976	0.000
Diameter—2nd follow-up visit (cm)	0.624 ± 0.0961		0.415 ± 0.0935	0.000

1st follow-up visit—3 months following surgery; 2nd follow-up visit—12 months following surgery.

**Table 3:** Receiver operating characteristic (ROC) curves for area and diameter.

Test Result Variable(s)	AUC (*p*-Value)
Initial Area (cm^2^)	0.581 (0.319)
Initial Diameter (cm)	0.602 (0.207)
Area—1st follow-up visit (cm^2^)	0.908 (0.000)
Diameter—1st follow-up visit (cm)	0.967 (0.000)
Area—2nd follow-up visit (cm^2^)	0.896 (0.000)
Diameter—2nd follow-up visit (cm)	0.938 (0.000)

AUC—Area Under the Curve.

**Table 4:** Histological scores in the two treatment groups.

Variable	Cryotherapy	Control	*p*-Value (Mann-Whitney U)
Fibrosis—intraoperative	0 (1)	0 (2)	0.975
Fibrosis—1st follow-up visit	2 (1)	2 (0)	0.541
Fibrosis—2nd follow-up visit	2 (1)	1 (1)	0.092
Mononuclear (inflammatory) cell infiltrate—intraoperative	3 (1)	3 (1)	0.838
Mononuclear (inflammatory) cell infiltrate—1st follow-up visit	1(1)	1.5 (1)	0.003
Mononuclear (inflammatory) cell infiltrate—2nd follow-up visit	0 (1)	1 (0)	0.000
Edema—intraoperative	2 (0)	2 (1)	0.446
Edema—1st follow-up visit	0.5 (1)	1 (1)	0.000
Edema—2nd follow-up visit	0 (1)	1 (1)	0.040
Ciliated cells—intraoperative	1 (0)	1 (1)	0.091
Ciliated cells—1st follow-up visit	2 (1)	1 (1)	0.007
Ciliated cells—2nd follow-up visit	2 (1)	1 (1)	0.000
Goblet cells—intraoperative	1 (1)	1 (1)	0.055
Goblet cells—1st follow-up visit	0 (1)	1 (1)	0.001
Goblet cells—2nd follow-up visit	0 (0)	1 (1)	0.000
Epithelial hyperplasia—intraoperative	2 (1)	2 (1)	0.273
Epithelial hyperplasia—1st follow-up visit	1 (2)	2 (1)	0.030
Epithelial hyperplasia—2nd follow-up visit	0 (1)	1 (0)	0.001
Squamous metaplasia—intraoperative	1 (1)	1 (1)	0.937
Squamous metaplasia—1st follow-up visit	1 (1)	1 (1)	0.082
Squamous metaplasia—2nd follow-up visit	0 (1)	1 (1)	0.069

1st follow-up visit—3 months following surgery; 2nd follow-up visit—12 months following surgery.

**Table 5:** The inflammatory parameter evolution in the SC group: Kendall’s correlation coefficient.

Histologic Parameter vs. Treatment Group	Kendall’s Tau-b	*p*-Value
Fibrosis—intraoperative	−0.004	0.975
Fibrosis—1st follow-up visit	−0.081	0.535
Fibrosis—2nd follow-up visit	0.221	0.072
Mononuclear (inflammatory) cell infiltrate—intraoperative	0.027	0.837
Mononuclear (inflammatory) cell infiltrate—1st follow-up visit	−0.391	0.000
Mononuclear (inflammatory) cell infiltrate—2nd follow-up visit	−0.510	0.000
Edema—intraoperative	0.102	0.438
Edema—1stfollow-up visit	−0.543	0.000
Edema—2nd follow-up visit	−0.279	0.033
Ciliated cells—intraoperative	−0.228	0.075
Ciliated cells—1st follow-up visit	0.358	0.002
Ciliated cells—2nd follow-up visit	0.488	0.000
Goblet cells—intraoperative	−0.262	0.030
Goblet cells—1st follow-up visit	−0.422	0.000
Goblet cells—2nd follow-up visit	−0.508	0.000
Epithelial hyperplasia—intraoperative	−0.144	0.260
Epithelial hyperplasia—1st follow-up visit	−0.284	0.019
Epithelial hyperplasia—2nd follow-up visit	−0.454	0.000
Squamous metaplasia—intraoperative	−0.011	0.937
Squamous metaplasia—1st follow-up visit	−0.232	0.065
Squamous metaplasia—2nd follow-up visit	−0.250	0.055

1st follow-up visit—3 months following surgery; 2nd follow-up visit—12 months following surgery.

**Table 6:** Correlation between inflammatory histological parameters and size of the middle meatal antrostomy (Spearman correlation test).

	Area	Diameter
Inflammatory cell infiltrate	r = −0.402 *p* = 0.040	r = −0.428 *p* = 0.029
Edema	r = −0.457 *p* = 0.012	r = −0.477 *p* = 0.003
Goblet cells	r = −0.437 *p* = 0.027	r = −0.469 *p* = 0.006
Epithelial hyperplasia	r = −0.475 *p* = 0.005	r = −0.452 *p* = 0.031

r—correlation coefficient.

**Table 7:** Correlation between inflammatory histological parameters (Spearman correlation test).

	Edema	Epithelial Hyperplasia
Inflammatory cell infiltrate	r = 0.410 *p* = 0.037	r = 0.425 *p* = 0.030
Goblet cells	r = 0.440 *p* = 0.022	r = 0.450 *p* = 0.012

r—correlation coefficient.

**Table 8:** Subjective outcomes in cryotherapy and control groups.

	Control Side	Cryotherapy Side	*p*-Value Student *t*-Test
	Mean ± SD	Mean ± SD	
Obstruction	7.53 ± 1.63	7.37 ± 1.72	0.36
Obstruction—2nd follow-up visit	3.18 ± 0.89	1.78 ± 0.72	0.000
Discharge	7.23 ± 1.65	7.10 ± 1.84	0.44
Discharge—2nd follow-up visit	3.43 ± 0.59	1.76 ± 0.73	0.000
Facial pressure	6.48 ± 1.72	6.51 ± 1.68	0.52
Facial pressure—2nd follow-up visit	2.1 ± 0.84	2.0 ± 0.75	0.38

2nd follow-up visit—12 months following surgery.

**Table 9:** Visual assessments in the preoperative and postoperative period.

	Preoperative	Postoperative Control	Postoperative Cryotherapy
Periorbital pain	No	No	No
Periorbital swelling	No	No	No
Proptosis	No	No	No
Globe displacement	No	No	No
Decreased ocular motility	No	No	No
Diplopia	No	No	No
Decreased color perception	No	No	No
Decreased visual acuity	No	No	No
Visual field loss	No	No	No
